# Acute aortic dissection in a young man with bilateral Covid-19 pneumonia: A suggestive case report at Computed Tomography Angiography

**DOI:** 10.1016/j.radcr.2022.01.034

**Published:** 2022-02-14

**Authors:** Francesco Messina, Lorena Turano, Grazia Calabrese, Carmela Tebala, Nicola Arcadi

**Affiliations:** Radiology Unit of Riuniti Hospital, Azienda Ospedaliera Grande Ospedale Metropolitano (G.O.M.) “Bianchi-Melacrino-Morelli”, Via Giuseppe Melacrino n.21, Reggio Calabria 89124, Italy

**Keywords:** Covid-19, Pneumonia, Computed Tomography Angiography, Dissection, Flap

## Abstract

Acute Aortic Dissection (AAD) is one of the most common lifethreatening diseases that affects the aortic vessel. An its immediate and accurate diagnosis is crucial to initiate the appropriate treatment.

The Covid-19 Coronavirus infectious pandemic started since December 2019 and was declared a pandemic by the World Health Organization in March 2020. It caused mainly bilateral interstitial pneumonia, up to causing a severe respiratory failure for the patients, and other complications.

Now, we describe the case of a young man that was admitted to our hospital and was found positive for the Coronavirus disease 2019 (Covid-19). While we were performing Computed Tomography (CT) scan of the chest, we had suspected the concomitant presence of an aortic dissection, which was then immediately confirmed by Computed Tomography Angiography (CTA) study, that we had performed to complete the baseline CT scan.

## Case presentation

A 52-years-old man with a medical history of hypertension and hyperlipidemia, was admitted to the Emergency Department of our Hospital with central chest pain of 2 days’ duration. The pain was described as sharp, radiating to the left arm, and associated with shortness of breath and fever (38°C). He recently had a contact with a people positive at Covid-19.

At Emergency Department the physical examination showed a pulseless right femoral artery, an arterial pressure of 170/85 mmHg, and a pulse rate of 70 beats/min. At the auscultation of thorax, there were pulmonary rales at the bases of both lungs, and an aortic diastolic murmur was detected during cardiac auscultation. Furthermore, in the laboratory tests there were increased values of PCR and procalcitonin. Oro-pharyngeal swab was positive for severe acute respiratory syndrome coronavirus 2 (SARS-CoV-2) by RT-PCR.

**Fig. 1:**
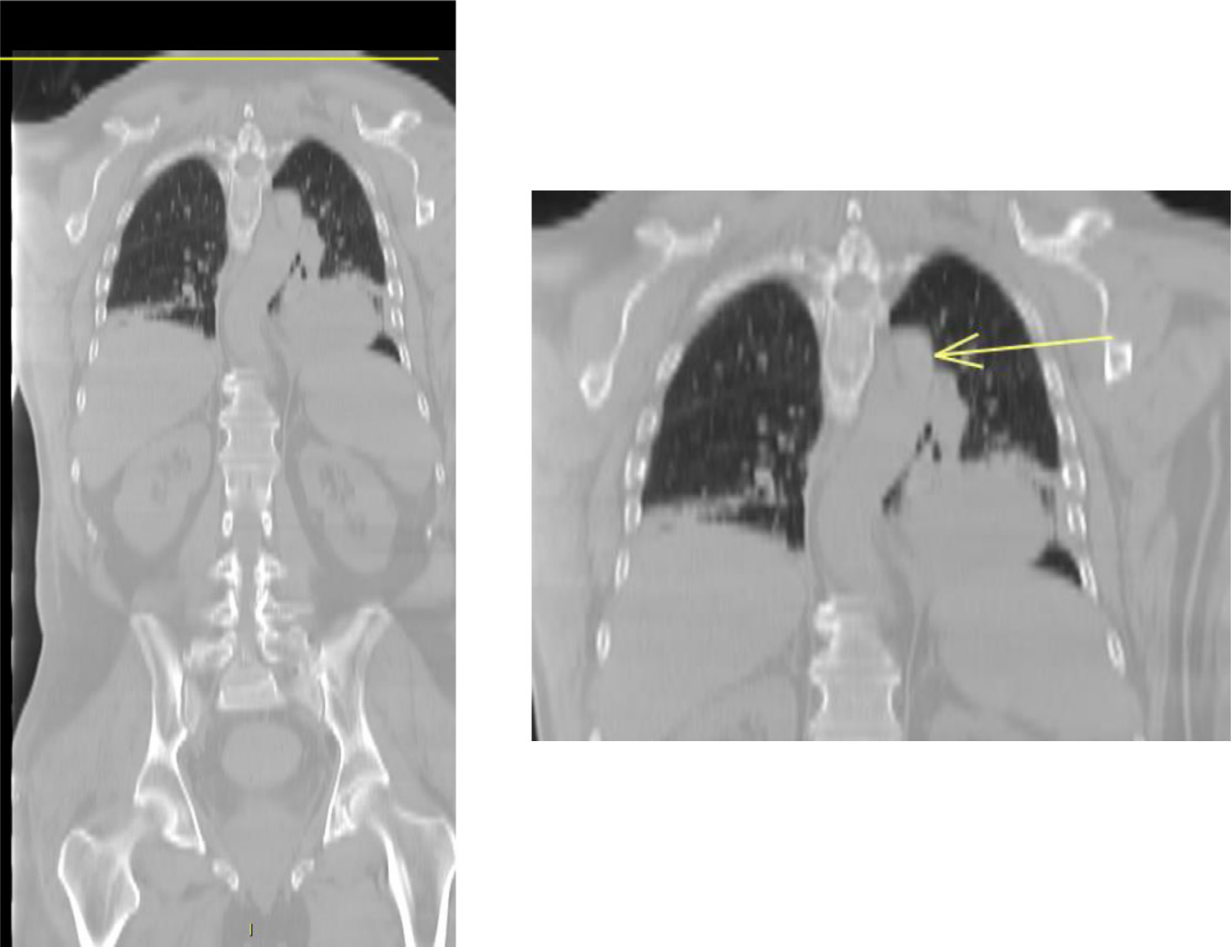
(A, B) Basal Chest High Resolution Computed Tomography (HRCT); coronal MPR reconstructions. HRCT had identified in the basal areas of both lungs the presence of thickenings with a ``ground glass'' pattern, and areas of interstitial consolidations and thickenings. There were also the presence of a minimal pleural effusion, and a lot of pericardial effusion. HRCT had also identified the presence, in basal conditions, of a thin intraluminal hypodense line in the thoracic aorta (yellow arrow), which raised the clinical suspicion of an aortic dissection.

Immediately, as a consequence of the patient's positivity to the molecular swab for Covid-19, a chest CT scan was urgently performed, in basal conditions and high resolution algorithm (HRCT), with a 64 ms multidetector scanner, and the images so obtained were analyzed with a slice-thickness of 1.2 mms and MPR reconstructions (axial, sagittal, and coronal). Basal HRCT had documented (Figs. 1A and B): in both lungs, in the basal areas, the presence of thickenings with a ``ground glass'' pattern, and areas of interstitial consolidations and thickenings. There were also the presence of a minimal pleural effusion, and a lot of pericardial effusion. But basal HRCT had also identified the presence of a thin intraluminal hypodense line in the thoracic aorta (yellow arrow), which raised our clinical suspicion of an aortic dissection.

For this reason, and the patient's symptoms and clinical situation, we immediately had decided to complete the HRCT with a contrast exam, as a Computed Tomography Angiography (CTA), to study the thoracic and abdominal aorta.

**Fig. 2:**
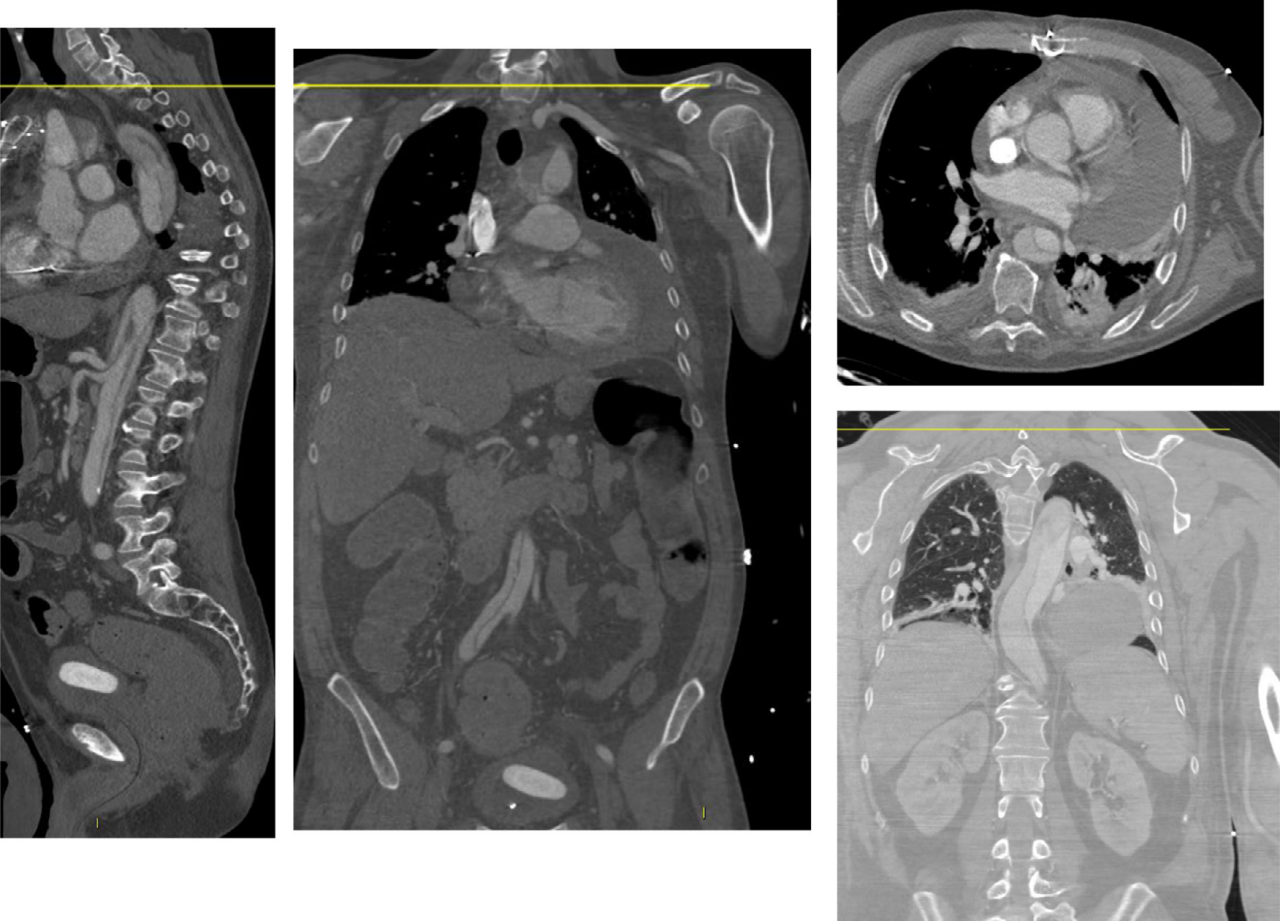
(A-D) Computed Tomography Angiography of the thoracic and abdominal cavities; sagittal, coronal and axial MPR reconstructions. Computed Tomography Angiography had confirmed the presence of a thin intraluminal hypodense line (intimal flap) in the thoracic aorta, and so had identified the presence of a suggestive dissection of the thoracic aorta at the ascending-arch passage which extends caudally to the iliac bifurcation and right iliac artery to its bifurcation, involving entirely the abdominal aorta for its entire course. It was also identified a pericardial effusion layer with a maximum thickness of about 5 cm (at the anterior pericardial wall).

CTA of thoracic-abdominal aorta had documented (Figs. 2A-D): the presence of a suggestive dissection of the thoracic aorta at the ascending-arch passage which extends caudally to the iliac bifurcation and right iliac artery to its bifurcation, involving entirely the abdominal aorta for its entire course.

The thoracic aorta in correspondence of the arch had a maximum diameter of about 35 mms with a true lumen of about 17 mms; in correspondence with the descending section the maximum diameter is about 30 mms with a true lumen of about 17 mms.

The abdominal aorta, with its suprarenal seat, had a maximum diameter of about 30 mms and a true lumen of about 14 mms. The celiac tripod and the superior mesenteric artery originate from the true lumen as well as the left renal artery. There is a double renal artery on the right, respectively with 1 origin from the false lumen and a "knightly" between the true lumen and the false lumen. It was also identified a pericardial effusion layer with a maximum thickness of about 5 cm (at the anterior pericardial wall).

The patient was immediately hospitalized, but due to the positive result and hemodynamic stability, cardiac surgery was postponed with the patient under intensive monitoring and therapeutic approach for Covid-19 pneumonia, and mobilization's restriction, in the Covid-19 designated intensive care unit of our hospital. Two weeks later and after negative tests for Covid-19, the patient underwent cardiac surgery to repair the aortic dissection, and after a total of about thirty days in our hospital, the patient had returned at home.

## Discussion

Acute aortic dissection (AAD) is one of the most potentially fatal pathologic processes within the aortic wall, which should be suspected in all patients presenting with acute chest or back pain. There are 2 classifications for AAD: Stanford and DeBakey. Stanford type A lesions involve the ascending aorta with or without extension to the descending aorta, whereas Stanford type B lesions are confined only to the descending aorta [1].

The Covid-19 infection strongly affects not only the respiratory system but also all of the systems in the body. The prognosis of the virus is worse in the elderly and in patients with chronic diseases, such as arterial hypertension, diabetes, obesity, chronic obstructive pulmonary disease, and asthma, and poses a higher risk for morbidity and mortality. In addition, Covid-19 infection may cause exacerbation of existing chronic conditions or acute and chronic complications of the cardiovascular system. More attention and awareness are thus needed on the acute and chronic effects of viral infection on the cardiovascular system. AAD, longitudinal cleavage of vessel media by blood, is commonly associated with a high mortality complication of a chronic cardiovascular pathology, which is frequently observed in patients with chronic complicated hypertension. As presented by Silvestri and colleagues [2] in their review of case reports, HIV-positive patients have a higher prevalence of aortic diseases, such as aortitis, aneurysms, and dissections, compared with patients without AIDS who have chronic cardiovascular diseases such as arterial hypertension. Reports are presented to evaluate the possibility of “similarity” of virulence as well as its therapy for HIV and SARS-CoV-2 and their infections [3].

The identification and management of aortic dissection in early stages is challenging because of its diverse presentation and life-threatening mimics, including acute coronary syndrome, pulmonary embolism, and stroke [4]. In the Covid-19 pandemic, a couple of factors have been hypothesized to increase the mortality of aortic dissection, including patients' fear of contracting Covid-19 and delayed presentation to the emergency department, super-saturated emergency departments, and delayed transfer by the emergency medical services. Handover in the emergency department is critical, playing a dynamic role in patient care; any lack of information can lead to compromise in quality of care provided. High clinical suspicion of Covid-19 based on suggestive history and findings along with painless presentation contributed to the difficulty in diagnosing AAD in the first place, and missing information during handover resulted in further delayed involvement of cardiothoracic surgery. Anchoring to the information received is important, but re-evaluating and going through details closely is also vital to avoid any anchoring bias that could have happened in this our case, but systematic approach and multidisciplinary practice led to diagnosis of AAD. CT has quickly become a cornerstone in both the diagnostic workup and follow-up of SARS-CoV-2 infection and is usually performed unenhanced. Immediate and accurate diagnosis of AAD is imperative and extremely important.

HRCT of the thorax is useful in early diagnosis of Covid-19 infection, in monitoring disease progression, coinfection, or disease stability. Computed Tomography had the possibility to evaluate accurately the type and extent of lung lesions. In our experience, we found that the most common CT findings were: GGO, consolidations, crazy-paving patterns, and pleural effusion. Consolidation was significantly more frequent in severe/critical patients, which indicates that the alveoli are completely filled by inflammatory exudation; this usually means that the virus diffuses into the respiratory epithelium, leading to necrotizing bronchitis and diffused alveolar damage [5].

## Conclusions

The Covid-19 pandemic had changed medical practices worldwide and had put severe stress on the healthcare. In this phase of adaptation, life-threatening diseases need special attention and care. Since aortic dissection is one of the life-threatening diseases with multiple variants, special attention must be given to atypical presentation, evolving triage criteria, and overlapping Covid-19 features which can further complicate and delay early identification and management. Computed Tomography had an important role for the diagnosis and evaluation of the severity of these diseases, because it investigates very well the dynamic CT changes in different stages of Covid-19 pneumonia, and also for the diagnosis and the best outcome/management of the patients with AAD.

## Patient consent

The patient confirmed the consense for publication of this case report.
